# Factors Associated With Non-adherence to Anti-depressant Medication in Adults: A Systematic Review and Meta-Analysis

**DOI:** 10.7759/cureus.37828

**Published:** 2023-04-19

**Authors:** Nazar Muhammad, Salecah R Ullah, Talwinder K Nagi, Rao Ahmed Yousaf

**Affiliations:** 1 Psychiatry, Cornerstone Family Healthcare, New York, USA; 2 Internal Medicine, Khawaja Muhammad Safdar Medical College, Sialkot, PAK; 3 Internal Medicine, Florida Atlantic University Charles E. Schmidt College of Medicine, Boca Raton, USA; 4 Psychiatry, Faisalabad Medical University, Faisalabad, PAK

**Keywords:** meta-analysis, depression, adults, anti-depressants, factors

## Abstract

The present meta-analysis has been conducted to review currently available literature to examine the factors associated with adherence to anti-depressant medications in adults. This meta-analysis and systematic review followed the MOOSE (Meta-analysis of Observational Studies in Epidemiology) guidelines. According to this analysis, the three most important electronic resources for research were CINAHL, EMBASE, and Medline. Google Scholar was used to supplementing the articles already available for review. Keywords used to find relevant articles included "predictors," "non-adherence," "anti-depressants," and "adults." Medical subject headings (MeSH) terms and Boolean operators ("AND" and "OR") were used in the search strategy to refine the search further. Studies included in this meta-analysis had information on factors associated with non-adherence to anti-depressant medication. The study evaluated samples of adult participants over 18 years with a diagnosis of depression and who had been prescribed anti-depressants.

In conclusion, this meta-analysis examined the relationship between demographic factors and non-adherence to anti-depressant medications. The findings revealed that gender, educational status, income level, marital status, and area of residence did not significantly predict non-adherence to anti-depressants. However, older age and polypharmacy were significant predictors of adherence to anti-depressants. The study also found that individuals living in urban areas were more likely to adhere to anti-depressants, but the difference was not statistically significant.

## Introduction and background

Due to its related mental, social, and interpersonal incapacity, depression has become a severe public health concern with a growing prevalence and worldwide disease burden [1]. Major depressive disorder (MDD) significantly burdens the global healthcare system regarding health-related quality of life, medical morbidity and mortality, and increased use of healthcare services [2]. An estimated 3.8% of the population experience depression, including 5% of adults (4% among men and 6% among women) and 5.7% of adults older than 60. Approximately 280 million people in the world have depression [3]. Depression is a common mental disorder, and it is characterized by persistent sadness and a lack of interest or pleasure in previously rewarding or enjoyable activities. The effects of depression can be long-lasting or recurrent and can dramatically affect a person's ability to function and live a rewarding life [4].

Due to its great prevalence, underdiagnosis, and inadequate treatment, depression also places a significant economic burden on society [5]. A comprehensive evaluation of the depression-related cost of illness research showed that depression was linked to a significant rise in direct and indirect expenses. The recent Global Burden of Disease 2017 report has provided further evidence that the crisis must be resolved quickly since it affects both public health and the mental health agenda [6]. The cornerstone of treatment for depressive illnesses is anti-depressant medication. Although many individuals with depressive disorders take prescription anti-depressants, they do not experience full remission, and over half of these patients experience recurring episodes over time. Strict adherence to a treatment plan is necessary to prevent the recurrence of depression [7].

The most popular and efficient treatments for depression are anti-depressant medications [8]. Even though numerous efficient anti-depressants are available, nearly 50% of individuals cannot eliminate symptoms and some experience recurrence. As a result, depression frequently develops into a chronic condition in patients and may require lifetime anti-depressant therapy [9]. Adherence to anti-depressant medication is essential for the intended treatment outcome. Non-adherence has been identified as the main issue with anti-depressant therapy. Adherence has been characterized as the degree to which a person complies with advice from a healthcare professional regarding taking medication, adhering to a diet, or leading a lifestyle-those who disregard medical advice risk relapsing and have a lower quality of life [9].

Treatment non-adherence can take many forms, including discontinuing therapy before the intended outcomes have been reached, failing to consistently attend scheduled therapy sessions, failing to follow prescription requirements, and failing to take prescribed medications [10]. Major personal and societal expenses are associated with treatment non-adherence. Non-adherence on a personal level adversely affects the quality of life, everyday functioning, and capacity for self-care of a depressed individual. The state of one's mental health may deteriorate as a result of non-adherence, and depression may recur. On a social level, non-adherence to treatment is linked to higher costs, primarily because of indirect costs like lost production through absenteeism and early retirement [9].

Much research is targeted at identifying predictors of non-adherence to suggest potential remedies due to the prevalence of depression treatment non-adherence and its detrimental effects [11]. Nevertheless, only a few variables have been found to influence adherence to depression treatment. Factors contributing to non-adherence must be identified to address them in interventions promoting the best possible medication use. Understanding the factors affecting medication adherence to anti-depressant medications will help policymakers and healthcare professionals design strategies to promote medication adherence to enhance the quality of life of these individuals and prevent the recurrence of depressive disorders. Therefore, the present meta-analysis has been conducted to review the currently available literature to examine the factors associated with adherence to anti-depressant medications in adults.

## Review

Methodology

This meta-analysis and systematic review followed the MOOSE (Meta-analysis of Observational Studies in Epidemiology) guidelines.

Search Strategy

According to this analysis, the three most important electronic resources for research were CINAHL, EMBASE, and Medline. Google Scholar was used to supplementing the articles already available for review. Keywords used to find relevant articles included "predictors," "non-adherence," "anti-depressants," and "adults." Medical subject headings (MeSH) terms and Boolean operators ("AND" and "OR") were used in the search strategy to refine the search further. These components were the foundation for creating search strings that accurately identify the finest articles. Identified articles were examined to find the most relevant for this study. The reference list of all included articles was also manually searched to ensure all articles in the current meta-analysis were accounted for. The search was conducted by two authors independently, and any disagreement between the two authors during the search was resolved through discussion.

Eligibility Criteria, Study Selection, and Quality Assessment

Studies included in this meta-analysis had information on factors associated with non-adherence to anti-depressant medication. The study evaluated samples of adult participants aged over 18 years with a diagnosis of depression and who had been prescribed anti-depressants. We excluded case reports, case series, editorials, and reviews. Only English-published articles were included in this meta-analysis. Two authors independently reviewed all eligible studies. After removing duplicates, initial screening was done using abstracts and titles, followed by a full-text screening of eligible studies. Any disagreement in the process of study selection was resolved through discussion. The quality of included studies was assessed using the National Institutes of Health (NIH) study quality assessment tools for the quality assessment of Observational Cohort and Cross-Sectional Studies.

Statistical Analysis

The investigation was conducted using two forms of analysis. The systematic review used a qualitative assessment, whereas the meta-analysis used a quantitative assessment. The systematic review also employed a literal analysis of the included studies' evidence. We then employed Review Manager Version 5.4.1 (RevMan 5.4.1: The Nordic Cochrane Centre, The Cochrane Collaboration) to perform the meta-analysis. The effect size was calculated using a random-effects model. The 95% confidence interval (CI) for the random-effects odds ratio (OR) was determined, while the P-value indicated the significance level. The difference is only considered significant if the P-value is less than 0.05. The I2 statistic, which has a value that runs from 0% (perfect consistency) to 100% (complete inconsistency), was used to describe heterogeneity. A forest plot was used to present the meta-analysis findings, and a funnel plot was used to display the publication bias among the examined studies.

Results

**Figure 1:**
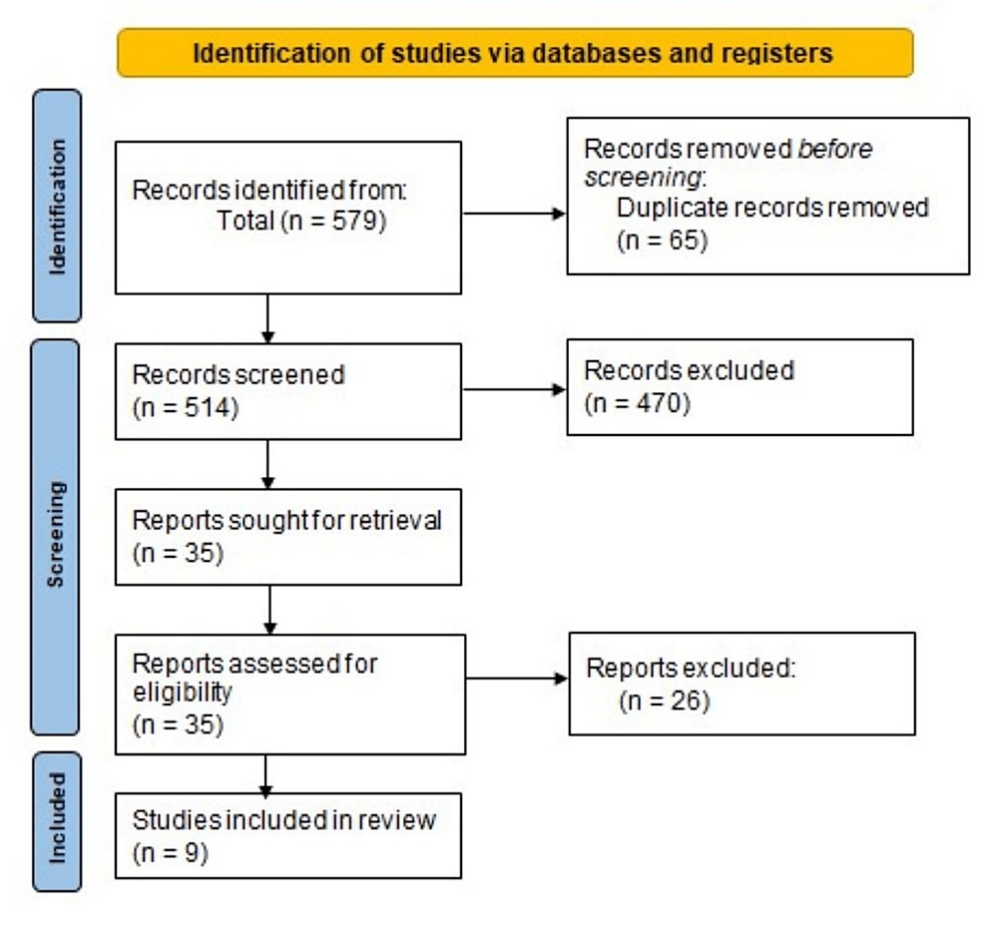
Flow diagram detailing the study selection process.

The online databases search resulted in 579 published studies. A search on PubMed and Medline generated the greatest number of studies. Of all these, however, 65 studies were eliminated due to duplication and other reasons for ineligibility employed through filtering. From that, 514 studies were screened for the title and abstract, where 479 were therefore exempted, leaving 35 articles. Of 35 studies, nine were included in this systematic literature review and meta-analysis. Figure 1 and Table 1 below represent a PRISMA flowchart diagram showing the included studies' selection process. Medication non-adherence ranged from 14.6% to 69.90%. The majority of the studies were crossectional (n=7). Three studies were conducted in the United States, two in India, and two in Spain. Table 2 shows a risk of bias in included studies.

**Table 1:** Characteristics of included studies MMAS: Morisky Medication Adherence Scale; MGLA; Morisky Green Levine Adherence; NR: Not reported

Study ID	Year	Study Design	Region	Total sample	Tool to measure adherence	Non-adherence (n)	Non-adherence (%)
Abegaz et al. [12]	2017	Cross-sectional	Ethiopia	217	MMAS	124	57.14
Alekhya et al. [13]	2015	Cross-sectional	India	103	Drug attitude inventory	72	69.90
Banerjee et al. [14]	2013	Cross-sectional	India	239	MMAS	160	66.95
Kales et al. [15]	2016	Cross-sectional	United States	311	Brief Medication Questionnaire	91	29.26
Marasine et al. [16]	2020	Cross-sectional	Nepal	174	MGLA	120	68.97
Pedrosa-Naudín et al. [17]	2022	Retrospective	Spain	246718	Medication possession ratio	49023	19.87
Serrano et al. [18]	2014	Cross-sectional	Spain	29	Drug attitude inventory	8	27.59
Slaughter et al. [19]	2012	Cross-sectional	United States	280	NR	41	14.64
Telinoiu [20]	2016	Retrospective	United States	626	Patient quality alliance	402	64.22

**Table 2:** Quality assessment of included studies. 1. Was the research question or objective in this paper clearly stated? 2. Was the study population clearly specified and defined? 3. Was the participation rate of eligible persons at least 50%? 4. Were all the subjects selected or recruited from the same or similar populations (including the same time period)? Were inclusion and exclusion criteria for being in the study prespecified and applied uniformly to all participants? 5. Was a sample size justification, power description, or variance and effect estimates provided? 6. For the analyses in this paper, were the exposure(s) of interest measured prior to the outcome(s) being measured? 7. Was the timeframe sufficient so that one could reasonably expect to see an association between exposure and outcome if it existed? 8. For exposures that can vary in amount or level, did the study examine different levels of the exposure as related to the outcome (e.g., categories of exposure, or exposure measured as continuous variable)? 9. Were the exposure measures (independent variables) clearly defined, valid, reliable, and implemented consistently across all study participants? 10. Was the exposure(s) assessed more than once over time? 11. Were the outcome measures (dependent variables) clearly defined, valid, reliable, and implemented consistently across all study participants? 12. Were the outcome assessors blinded to the exposure status of participants? 13. Was loss to follow-up after baseline 20% or less? 14. Were key potential confounding variables measured and adjusted statistically for their impact on the relationship between exposure(s) and outcome(s)? NA: Not applicable; NR: Not reported

Study ID	1	2	3	4	5	6	7	8	9	10	11	12	13	14	Overall
Abegaz et al. [12]	Yes	Yes	Yes	Yes	No	NA	Yes	No	Yes	No	NA	NA	NA	Yes	Fair
Alekhya et al. [13]	Yes	Yes	NR	Yes	No	NA	Yes	No	Yes	No	NA	NA	NA	No	Poor
Banerjee et al. [14]	Yes	Yes	Yes	Yes	No	NA	Yes	No	Yes	No	NA	NA	NA	No	Poor
Kales et al. [15]	Yes	Yes	Yes	Yes	No	NA	Yes	No	Yes	No	NA	NA	NA	Yes	Fair
Marasine et al. [16]	Yes	Yes	Yes	Yes	Yes	NA	Yes	No	Yes	No	NA	NA	NA	Yes	Good
Pedrosa-Naudín et al. [17]	Yes	Yes	Yes	Yes	No	NA	Yes	No	Yes	Yes	NA	NA	NA	Yes	Good
Serrano et al. [18]	Yes	Yes	NR	Yes	No	NA	Yes	No	Yes	No	NA	NA	NA	No	Poor
Slaughter et al. [19]	Yes	Yes	NR	Yes	No	NA	Yes	No	Yes	No	NA	NA	NA	Yes	Fair
Telinoiu [20]	Yes	Yes	NR	Yes	No	NA	Yes	No	Yes	No	NA	NA	NA	Yes	Fair

Predictors of Non-adherence to Antidepressant Medications

**Figure 2:**
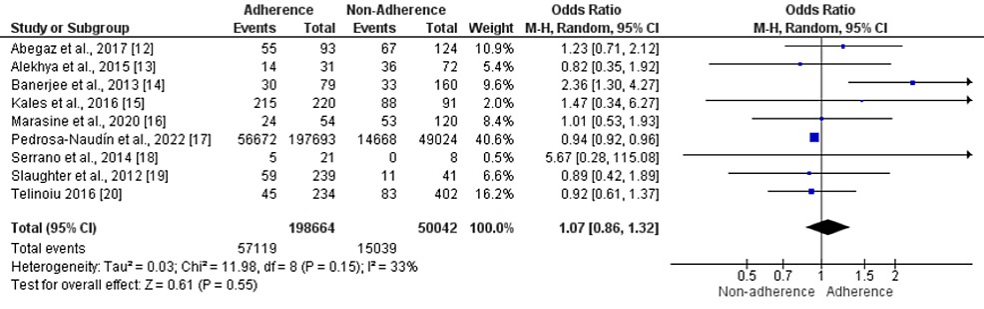
Effect of Gender. An odds ratio>1 shows a high number of males in the Adherence group, while an odds ratio<1 shows a high number of males in the non-adherence group. Sources: References [12-20]

Gender: Nine studies examined gender as a predictor of non-adherence, and most did not show any relationship between gender and non-adherence to anti-depressant medications. The pooled analysis showed that the number of males in the adherence group was higher, but the difference was statistically insignificant (OR: 1.07, 95% CI: 0.86-1.32), as shown in Figure 2. Moderate heterogeneity was reported among the study results (I-square: 33%).

**Figure 3:**
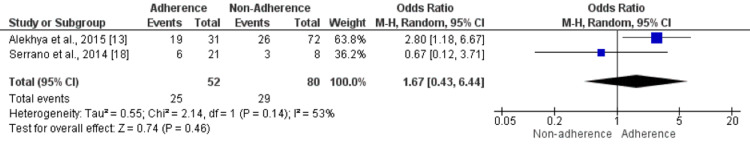
Effect of educational status A odds ratio>1 shows a high number of individuals with no education or primary education in the Adherence group. In contrast, an odds ratio<1 shows a high number of individuals with no education or primary education in the non-adherence group. Sources: References [13,18]

Educational Status: Five of the included studies examined the effect of educational status on non-adherence to anti-depressant medications. Two studies in the pooled analysis showed no significant difference in the odds of no education or primary education between patients who adhered to and non-adhered medications (OR: 1.67, 95% CI: 0.43-6.44), as shown in Figure 3. Moderate heterogeneity was reported among the study results (I-square: 53%). A study conducted by Slaughter [19], Marasine et al. [16], and Banerjee [14] did not demonstrate any significant impact of educational status on non-adherence to anti-depressant medications.

**Figure 4:**
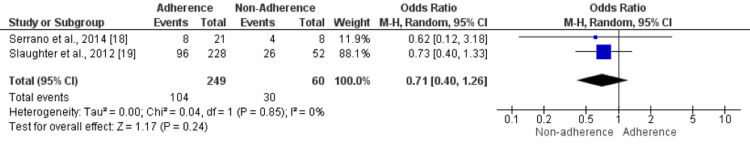
Effect of income. An odds ratio>1 shows a high number of individuals with low income in the Adherence group. In contrast, an odds ratio<1 shows a high number of individuals with low income in the non-adherence group. Sources: References [18-19]

Income Level: A pooled analysis of two studies showed no significant effect of income level on non-adherence to anti-depressant medications. The odds of lower income were not significantly different in patients who adhered to and patients who did not adhere to anti-depressant medications (OR: 0.71, 95% CI: 0.40-1.26), as shown in Figure 4. No heterogeneity was reported among the study results. None of the included studies showed a significant income level impact on non-adherence to anti-depressant medications.

**Figure 5:**
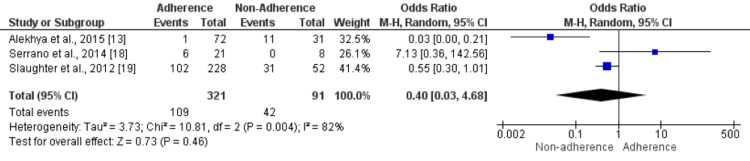
Effect of Marital Status. An odds ratio>1 shows a high number of individuals who never married in an Adherence group. In contrast, an odds ratio<1 shows a high number of individuals who never married in a non-adherence group. Sources: References [13,18,19]

Marital Status: Three studies were included in the pooled analysis to determine the effect of marital status on non-adherence to anti-depressant medications. The odds of being unmarried were not significantly different in patients who adhered to and did not adhere to anti-depressant medications (OR: 0.40, 95% CI: 0.03-4.68), as shown in Figure 5. High heterogeneity was reported among the study results (I-square: 82%). Of the three studies, only one showed a significant impact of marital status on non-adherence to anti-depressant medications.

**Figure 6:**
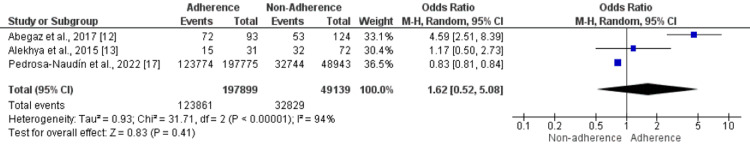
Effect of Area of Residence. An odds ratio>1 shows a high number of individuals who live in urban areas in the Adherence group. In contrast, an odds ratio<1 shows a high number of individuals who live in urban areas in the non-adherence group. Sources: References [12,13,17]

Area of Residence: Three studies were included in the pooled analysis to determine the effect of area of residence on non-adherence to anti-depressant medications. The odds of living in an urban area are insignificant in patients who adhered to and those who did not adhere to anti-depressant medications (OR: 1.62, 95% CI: 0.52-5.08), as shown in Figure 6. High heterogeneity was reported among the study results (I-square: 94%). Out of the three included studies, one study showed that the odds of urban were significantly higher in the non-adherence group [17], while another showed contrasting results [12]. The study by Alekhya et al. showed no significant difference [13].

**Figure 7:**
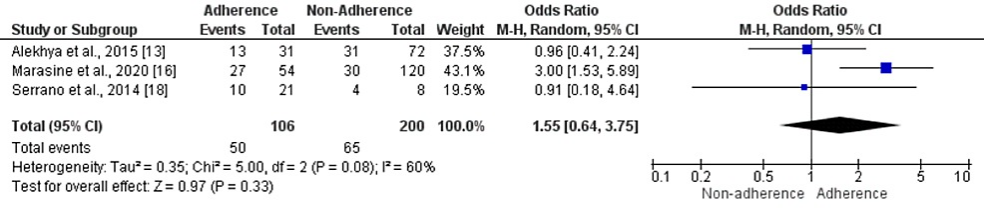
Effect of employment status. An odds ratio>1 shows a high number of individuals who were employed in the Adherence group. In contrast, an odds ratio<1 shows a high number of individuals who were employed in the non-adherence group. Sources: References [13,16,18]

Employment Status: Three studies assessed the impact of employment on non-adherence to anti-depressant medications. The study found that the odds of being employed were not significantly different in patients who adhered to medications compared to their counterparts (OR: 1.55, 95% CI: 0.64-3.75), as shown in Figure 7. Moderate heterogeneity was reported among the study results (I-square: 60%).

**Figure 8:**
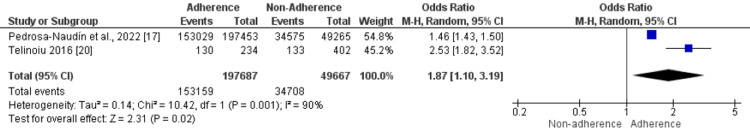
Effect of polypharmacy. An odds ratio>1 shows a high number of individuals with polypharmacy in the Adherence group. In contrast, an odds ratio<1 shows a high number of individuals with polypharmacy in the non-adherence group. Sources: References [17,20]

Polypharmacy: Two studies assessed the impact of employment on non-adherence to anti-depressant medications. The study found that the odds of polypharmacy were higher in patients who adhered to medications than in their counterparts (OR: 1.87, 95% CI: 1.10-3.19), as shown in Figure 8. High heterogeneity was reported among the study results (I-square: 90%).

Age: Six studies assessed the impact of age on non-adherence to anti-depressant medication. However, we did not perform quantitative pooling because the age cut-off in individual studies differed. We summarized that the youngest participants (usually aged < 50 years) were significantly more likely to have lower adherence. However, older adults (usually ≥ 70 years old) also displayed lower adherence.

Discussion

Anti-depressants have been questioned regarding their long-term efficacy and safety for thousands of patients [21,22]. However, they can be helpful for some patients by reducing depressive symptoms and lowering the likelihood of relapse. Adherence to anti-depressant medication is crucial for the effectiveness of the treatment and to avoid the reemergence of the symptoms and the disease in general. The present meta-analysis found only age and polypharmacy significant factors affecting non-adherence to anti-depressants.

Published research is abundant on variables that may contribute to poor anti-depressant adherence. Age is one-factor affecting anti-depressant drug non-adherence [13,23-24]. Older individuals have higher instances of not starting but lower rates of suboptimal anti-depressant adherence compared to younger adults [25]. This suggests that older individuals are less likely to initiate and accept treatment, but once they do, they are more likely to stick with it, resulting in high compliance rates. It is likely because, in developed countries, old-age adults are cared for by caregivers in nursing homes or old-age homes [17]. Therefore, it is important to note that access to healthcare services and resources can also significantly affect older adults' ability to initiate and adhere to treatment. Disparities in these areas can contribute to poorer health outcomes in this population.

Our meta-analysis identified polypharmacy as a predictor for preventing non-adherence to anti-depressants, and this contradicts the results of a comprehensive literature review [26]. In contrast to some research suggesting that taking multiple medications leads to non-adherence among older adults [27], most studies in our meta-analysis suggested that older age significantly contributes to anti-depressant adherence. Previous research has supported that the prevalence of polypharmacy in the elderly is increasing [28], especially in patients with mental disorders [29]. This is an important factor to consider because the number of prescribed medications and the complexity of the regimen is linked to lower medication adherence [30].

Our meta-analysis found that individuals living in urban areas are more likely to adhere to anti-depressants. However, the difference was statistically insignificant. All three studies in the pooled analysis showed contrasting results [12,13,17]. We initially anticipated that patients living in urban areas would have better adherence to anti-depressants than those living in rural areas, as the proximity to pharmacies is easier. However, studies favoring adherence in rural areas indicate that patients may have better adherence due to healthcare professionals having a higher workload and a closer relationship with individual patients. Despite this, previous studies have suggested that the location of residence, whether urban or rural, does not significantly impact medication adherence [31].

Concerning anti-depressants, there is growing evidence that older patients have unfavorable attitudes toward them, including worries about side effects and fear of addiction that compares anti-depressants to illicit narcotics [32]. According to studies, older patients perceive a much greater need for anti-depressant medication than younger ones [33]. Patients' belief systems significantly impact adherence to anti-depressant medications. Stigma and forgetfulness were not significant enough, although very few studies were used. Therefore, more analysis needs to be conducted in that regard. Initial factors promoting drug adherence were a person's attitude, awareness of their depressive state, and faith in anti-depressant medications. On the other hand, non-adherence to treatment was hampered by a negative attitude toward their depression and a lack of faith in anti-depressant drugs [34]. As a result, early education regarding depression and anti-depressant treatment should be emphasized to help people who have recently been diagnosed with depression and prescribed anti-depressant medications adhere to their medication regimens [35].

The present meta-analysis has limitations: High heterogeneity was reported among specific predictors. Heterogeneity is likely due to differences in regions, sample size, and tools used to assess medication adherence. We were not able to perform subgroup analysis due to a limited number of studies; Few studies in the present meta-analysis did not use a validated instrument to assess medication adherence; Certain factors were not assessed in the present meta-analysis due to insufficient available studies, including knowledge about side effects, caregiver role, etc.

The current predictors of adherence need to be used to identify high-risk populations for targeted counseling and to design interventions to promote adherence aimed at these high-risk individuals. Particular attention needs to be paid to younger individuals, and it is also important for healthcare professionals to consider non-pharmacological approaches to reduce the relapse of depression. Future studies need to be conducted to include sample size and use a validated tool to identify predictors of non-adherence to anti-depressant medications.

## Conclusions

In conclusion, this meta-analysis examined the relationship between demographic factors and non-adherence to anti-depressant medications. The findings revealed that gender, educational status, income level, marital status, and area of residence did not significantly predict non-adherence to anti-depressants. However, older age and polypharmacy were significant predictors of adherence to anti-depressants. The study also found that individuals living in urban areas were more likely to adhere to anti-depressants, but the difference was not statistically significant. These findings suggest that healthcare professionals should pay close attention to older patients and those taking multiple medications to ensure better anti-depressant adherence. Additionally, the study highlights the need for more research to identify factors contributing to medication adherence among patients with mental disorders.
